# Xyloketal B Attenuates Fatty Acid-Induced Lipid Accumulation via the SREBP-1c Pathway in NAFLD Models

**DOI:** 10.3390/md15060163

**Published:** 2017-06-03

**Authors:** Youying Zhang, Tian Meng, Ling Zuo, Yu Bei, Qihao Zhang, Zhijian Su, Yadong Huang, Jiyan Pang, Qi Xiang, Hongtu Yang

**Affiliations:** 1 Institute of Biomedicine & Guangdong Provincial Key Laboratory of Bioengineering Medicine, Jinan University, Guangzhou 510632, China; qq329174818@163.com (Y.Z.); mt4219466@163.com (T.M.); 15521371419@163.com (L.Z.); xiaobei7253483@163.com (Y.B.); tqhzhang@jnu.edu.cn (Q.Z.); tjnuszj@jnu.edu.cn (Z.S.); tydhuang@jnu.edu.cn (Y.H.); 2 Department of Pharmacy, Jinan University, Guangzhou 510632, China; yht2113@126.com; 3 School of Chemistry and Chemical Engineering, Sun Yat-Sen University, Guangzhou 510275, China; 4 The People’s Hospital of Shenzhen City, Shenzhen 518020, China

**Keywords:** Xyloketal B, NAFLD, SREBP-1c pathway

## Abstract

The goal of this study was to examine the effects of xyloketal B on nonalcoholic fatty liver disease (NAFLD) and to explore the molecular mechanisms underlying its effects in both in vivo and in vitro models. We discovered an association between xyloketal B and the sterol regulatory element-binding protein-1c (SREBP-1c) signaling pathway, which is related to lipid metabolism. Mice were dosed with xyloketal B (5, 10 and 20 mg/kg/d) and atorvastatin (15 mg/kg/d) via intraperitoneal injection once daily for 40 days after being fed a high fat diet plus 10% high fructose liquid (HFD+HFL) for 8 weeks. Xyloketal B significantly improved HFD+HFL-induced hepatic histological lesions and attenuated lipid and glucose accumulation in the blood as well as lipid accumulation in the liver. Xyloketal B increased the expression of CPT1A, and decreased the expression of SREBP-1c and its downstream targeting enzymes such as ACC1, ACL, and FAS. Xyloketal B also significantly reduced lipid accumulation in HepG2 cells treated with free fatty acids (FFAs). These data suggested that xyloketal B has lipid-lowering effects via the SREBP-1c pathway that regulate lipid metabolism. Thus, targeting SREBP-1c activation with xyloketal B may be a promising novel approach for NAFLD treatment.

## 1. Introduction

Nonalcoholic fatty liver disease (NAFLD) with no relation to an excessive drinking history is a clinicopathological syndrome of fat storage and steatosis in liver parenchyma cells [1,2]. NAFLD includes four pathological stages, beginning with simple fatty liver, followed by nonalcoholic steatohepatitis (NASH), liver fibrosis, and ultimately cirrhosis, hepatocellular cirrhosis, and hepatocellular carcinoma [3,4,5]. The pathogenesis of NAFLD is complicated and still requires clarification. Currently, the “two hits theory” proposed by James and Day is widely accepted as an explanation of the pathogenesis of NAFLD [6]. The first hit is mainly steatosis, which is primarily caused by insulin resistance and lipid metabolism disorder, and the second hit refers to oxidative stress and lipid peroxidation damage, which are caused by NASH. If NASH persists, an inflammatory necrosis cycle occurs, which then progresses to fatty liver fibrosis and fatty liver cirrhosis. In Western countries, NAFLD has become a common chronic liver disease with approximately 20% of the population suffering from this disease, and the rate of NAFLD in the obese population is 70%–80% [7,8,9]. In approximately 25% of patients with NASH, fibrosis of the hepatic portal vein [10] evolves into liver cirrhosis. To date, there is no effective drug for the treatment of NAFLD, and the biological mechanism underlying steatosis occurrence and progression to NAFLD is unclear [5,11]. Therefore, the present study focuses on the pathogenesis of NAFLD to help develop therapeutic drugs.

Sterol regulatory element-binding protein-1 (SREBP-1), an important lipogenic transcription factor, preferentially regulates many types of key lipogenic genes encoding enzymes involved in triglyceride synthesis and accumulation [12]. Enzymes downstream of SREBP-1, including ATP-citrate lyase (ACL), acetyl-CoA carboxylase (ACC1), and fatty acid synthase (FAS), are the key enzymes of de novo lipogenesis [13,14]. ACL catalyzes citrate into acetyl-CoA, and ACC1 dimerizes and decarboxylates acetyl-CoA into malonyl-CoA. FAS further elongates malonyl-CoA by adding two carbon fragments to form fatty acyl-CoA. Malonyl-CoA, a key intermediate of fatty acid synthesis, inhibits CPT1A and blocks β-oxidation, thus contributing to intrahepatic lipid accumulation [15,16]. Activation of SREBP-1c promotes the induction of such lipogenic genes, resulting in increased fatty acid synthesis and further progression to hypertriglyceridemia. Thus, the SREBP-1c signaling pathway has been regarded as a potential target for treating NAFLD.

Xyloketal B is isolated from the South China Sea marine fungus *Xylaria sp. (no. 2508)* [17] and is a novel benzopyran compound with a unique chemical structure [18,19,20]. Our previous studies have shown that xyloketal B has strong pharmacological properties such as L-calcium channel-blocking activity in hippocampal cells [21], neuroprotective activities in an MPP+-induced C. elegans Parkinson’s disease (PD) model [22], and antioxidative activity, which can attenuate NADPH oxidase-derived reactive oxygen species (ROS) generation in oxLDL-treated endothelial cells [23]. Moreover, xyloketal B can significantly protect human umbilical vein endothelial cells (HUVECs) from injury induced by ox-LDL, without proliferative or toxic effects in vitro [24]. A zebrafish study has also indicated that xyloketal B exhibits antioxidative and antiapoptotic abilities through the induction of HO-1 expression via the PI3K/Akt/Nrf-2 pathway [25]. In addition, xyloketal B has anti-atherosclerotic effects in APOE-deficient mice and can attenuate increasing blood pressure in hypertensive rats [19]. Atherosclerosis is characterized by lipid deposition in blood vessels, which causes vessel walls to thicken. The first hit in NAFLD pathogenesis mentioned above is due to the accumulation of lipid in the liver. Atherosclerosis and NAFLD are related to lipid metabolism disorder. Therefore, we hypothesize that xyloketal B will have a lipid-lowering effect and can be used to treat NAFLD.

Thus, in this study, we first characterized the preventive and therapeutic effects of xyloketal B in an NAFLD mouse model and then investigated the underlying mechanisms of xyoketal B’s effects on NAFLD progression.

## 2. Results

### 2.1. Xyloketal B Treatment Reduces Body Weight, Fat Weight, Liver Index, and Body Mass Index Without Influencing Food Intake

Xyloketal B showed a protective effect on body weight, fat weight, liver index (liver weight/body weight), and body mass index (BMI; body weight/height) in the mice. All treatment groups showed a significant reduction (*p* < 0.01) in fat weight, liver index, and BMI compared with the model group (Table 1). Thus, these data demonstrated that xyloketal B reduces the body weight, fat weight, hepatic index, and BMI of HFD+HFL-fed mice.

### 2.2. Xyloketal B Treatment Improves Hepatic Histology

**Figure 1:**
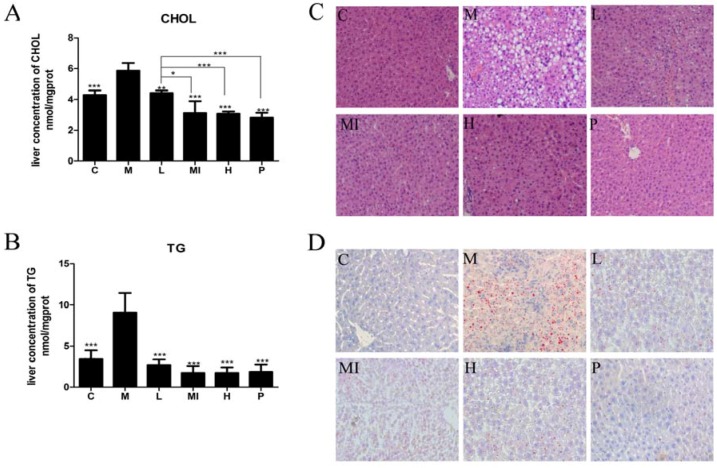
Xyloketal B ameliorates HFD+HFL-induced liver lipid accumulation. (**A**) Hepatic total cholesterol content after 40 days. (**B**) Hepatic triglyceride content after 40 days. (**C**) Hematoxylin and eosin staining after 40 days revealed fat accumulation in the liver (original magnification of 400×). (**D**) Intracellular lipid droplets in the hepar were stained with Oil Red O and observed at 400× magnification. C: control group; M: model group; L: 5 mg/kg/d xyloketal B group; Mi: 10 mg/kg/d xyloketal B group; H: 20 mg/kg/d xyloketal B group; P: 15 mg/kg/d atorvastatin group. Data are expressed as the means ± SEM (*n* = 8). * *p* < 0.05 and *** *p* < 0.01 versus the M group.

Liver histological analysis with H&E staining is the NAFLD diagnostic gold standard. According to the results of our study, the development of hepatic steatosis occurred in the mouse model. Liver sections from normal chow-fed mice (C group) had a normal morphological appearance. Liver sections from the HFD+HFL-fed mice (M group) showed a large number of diffuse fat vacuoles, a higher degree of macrovesicular steatosis, hepatocellular ballooning, and obvious inflammatory sites. Following xyloketal B treatment for 40 days, hepatic steatosis was markedly ameliorated in the different treatment groups (Figure 1C). The serum level of transaminase is a sensitive indicator of hepatic injury. After histological observation of the hepatic injury, we detected serum AST and ALT levels in each group of mice. After the 40-day study, the progression of NAFLD significantly increased the serum levels of AST and ALT compared with those in the control mice. Xyloketal B treatment alleviated hepatic injury by decreasing the levels of AST and ALT (Table 1).

### 2.3. Xyloketal B Ameliorates Lipid Accumulation

To observe the changes in fat and glucose levels in the blood, we detected serum levels of TG, CHOL, HDL-C, LDL-C, APOE, and GLU. In this study, the HFD+HFL-fed mice had higher serum levels of fasting blood glucose, TG, CHOL, HDL-C, and LDL-C than those in the control group, and mice treated with the different doses of xyloketal B showed a statistically significant reduction in these levels (Table 1). In addition, the different doses of xyloketal B significantly increased APOE levels (Table 1). Moreover, TG and CHOL accumulated in the livers of mice treated with HFD+HFL compared with the control group, but treatment with xyloketal B for 40 days decreased the TG and CHOL levels (Figure 1A,B). These results suggested that a high-fat and high-fructose diet can induce NAFLD and metabolic syndrome in mice. Furthermore, these results demonstrated that xyloketal B treatment prevents NAFLD progression.

### 2.4. Xyloketal B Reduces the Expression of Lipid Metabolism-Related Molecules

**Figure 2:**
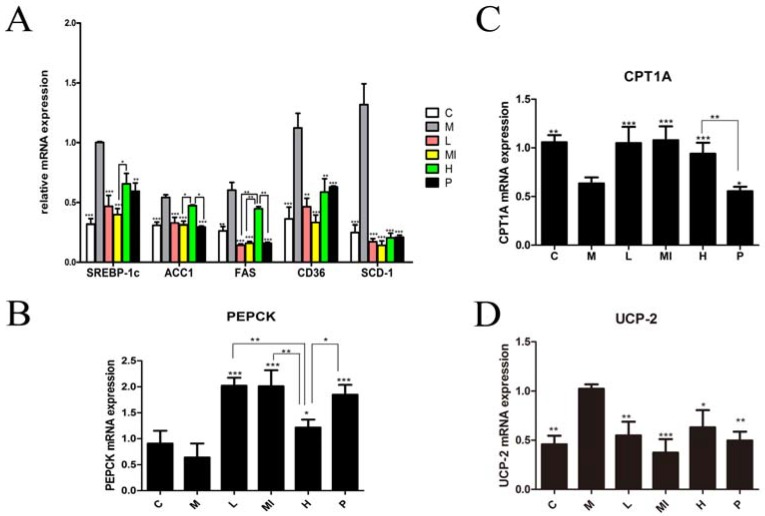
Gene expression in liver tissue following xyloketal B treatment for 40 days. Xyloketal B down-regulated the gene expression associated with de novo lipogenesis pathways. (**A**) mRNA expression of SREBP-1c and its downstream-targeting genes, including (**B**) PEPCK; (**C**) CPT1A and CPT1B; and (**D**) UCP-2. C: control group; M: model group; L: 5 mg/kg/d xyloketal B group; Mi: 10 mg/kg/d xyloketal B group; H: 20 mg/kg/d xyloketal B group; *p*: 15 mg/kg/d atorvastatin group. Data are expressed as the means ± SEM (*n* = 8).* *p* < 0.05, ** *p* < 0.01, and *** *p* < 0.001 versus the M group.

Previous research and the “two hits theory”, indicate that NAFLD has a close relationship with lipid metabolism disorders. To determine further effects of xyloketal B treatment on lipid metabolism disorders induced by HFD+HFL feeding, we analyzed the expression of genes and proteins involved in lipid metabolism using real-time PCR and Western blotting, respectively. The different xyloketal B dosage groups significantly down-regulated the mRNA expression of SREBP-1c, FAS, ACC1, SCD-1, and CD36 compared with the levels in the model group (Figure 2A). Furthermore, the levels of phosphorenol pyruvate carboxy kinase (PEPCK) and carnitine palmitoyl transferase 1 (CPT1) were significantly increased by xyloketal B treatment compared with those in the model group (Figure 2B,C). Uncoupling protein 2 (UCP-2), which is involved in metabolic disorders and oxidative stress, was remarkably decreased by xyloketal B treatment compared with the model group (Figure 2D). In addition, the experimental results showed that there was no dose dependence in the xyloketal B treatment groups.

**Figure 3:**
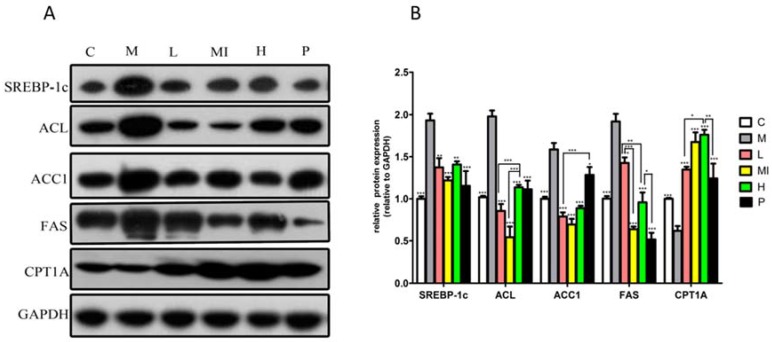
Western blot analysis of SREBP1-C, ACC1, FAS, ACL, and CPT1-A protein levels in mice liver treated with xyloketal B for 40 days. Representative immunoblots (**A**) and quantification (**B**) of the expression of proteins related to lipid metabolism in mice liver. C: control group; M: HFD+HFL group; L: 5 mg/kg/d xyloketal B group; Mi: 10 mg/kg/d xyloketal B group; H: 20 mg/kg/d xyloketal B group; P: 15 mg/kg/d atorvastatin group. Data are expressed as the means ± SEM (*n* = 8). * *p* < 0.05, ** *p* < 0.01, and *** *p* < 0.001 versus the M group.

Our study also demonstrated that the expression of lipid metabolism-related proteins significantly changed in the livers of mice subjected to xyloketal B treatment for 40 days. Groups treated with xyloketal B showed significant reductions in SREBP-1c, ACC1, FAS, and ACL protein expression, as well as increased CPT1A protein expression (Figure 3) compared with the levels in the model group. Thus, these results indicate that xyloketal B reduces hepatic lipid accumulation via the modulation of SREBP-1c and the de novo lipogenesis synthesis pathway. 

### 2.5. Xyloketal B Reduces Intracellular Lipid Accumulation via Activation of the SREBP-1c Signaling Pathway

**Figure 4:**
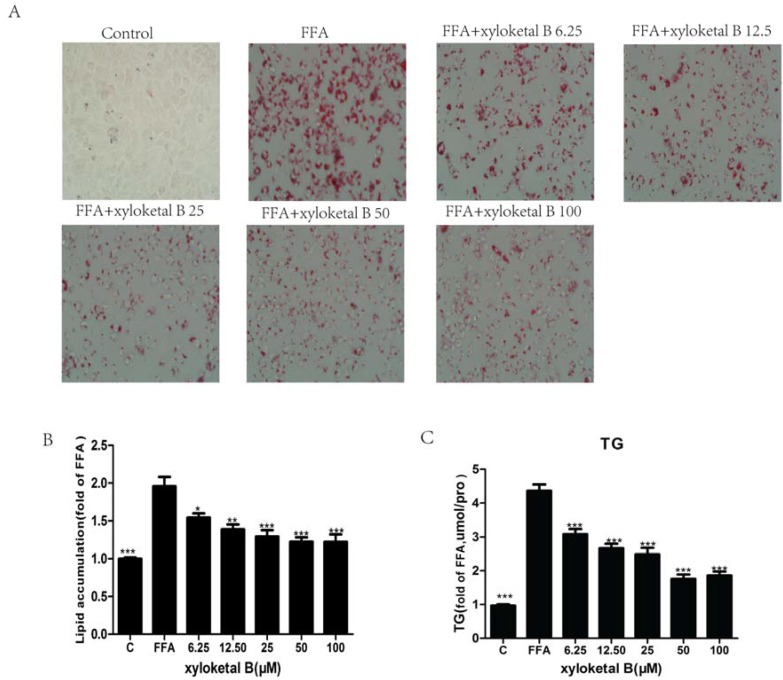
Xyloketal B-mediated effects on lipid accumulation and TG content in FFA-treated HepG2 cells. HepG2 cells were treated with FFA (0.165 mM PA and 0.33 mM OA) for 24 h and were then incubated with various concentrations of xyloketal B for 24 h. (**A**) Intracellular lipid droplets in HepG2 cells were stained with Oil Red O and observed at 400× magnification. (**B**) Lipid droplets were quantified by measuring the resultant absorbance at 540 nm. (**C**) Quantitative analysis of intracellular TG content in HepG2 cells. Data are presented as the means ± SEM (*n* = 3). * *p* < 0.05, ** *p* < 0.01, and *** *p* < 0.001 versus the FFA group.

To extend the in vivo results and confirm whether the hypolipidemic effects of xyloketal B were mediated by SREBP-1c, we employed HepG2 cells to verify our in vivo results using an in vitro model. Before proceeding with the subsequent experiment, we examined the effect of various concentrations of xyloketal B on HepG2 cell viability using the MTT assay. Cell viability was unaffected by treatment with xyloketal B at concentrations up to 50 μM, but cell viability was significantly reduced by treatment with 100 μM xyloketal B. Thus, xyloketal B concentrations ≤50 μM were used in this experiment. The intracellular lipid levels were significantly different between the control group and FFA group. The control group had almost no lipid droplets, whereas the FFA group had macro-lipid droplets and severe steatosis. Compared with the FFA group, the numbers and sizes of the lipid droplets were significantly less in the xyloketal B groups (Figure 4A). Furthermore, quantification of intracellular lipids revealed that xyloketal B treatment significantly improved steatosis induced by FFA in a dose-dependent manner (Figure 4B). The intracellular TG content measurements also showed that xyloketal B suppressed the lipid accumulation in a dose-dependent manner and exerted a significant inhibitory effect on HepG2 cells treated with FFA (Figure 4C). 

**Figure 5:**
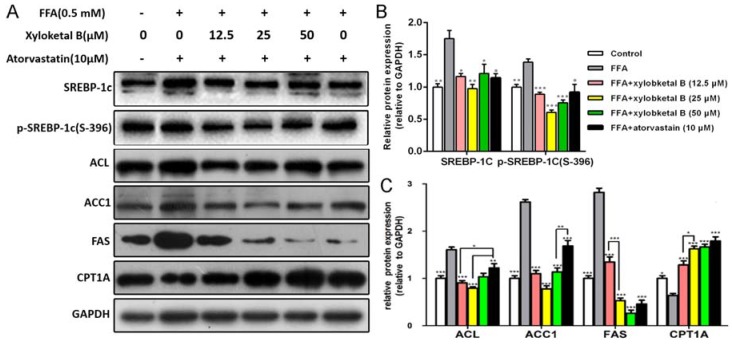
Effects of xyloketal B on the expression of proteins related to lipid metabolism in HepG2 cells. HepG2 cells were treated with xyloketal B (12.5, 25 or 50 μM) for 24 h. Representative immunoblots (**A**) and quantification (**B**), (**C**) of the expression of proteins related to lipid metabolism in HepG2 cells. The results are expressed as the means ± SEM of three independent experiments. * *p* < 0.05, ** *p* < 0.01, and *** *p* < 0.001 versus the FFA group.

To further study the effect of xyloketal B on lipid metabolism, we examined the protein expression of SREBP-1c, p-SREBP-1c (S-396), ACC1, ACL, FAS, and CPT-1A. As shown in Figure 5B, the protein expression of SREBP-1c and p-SREBP-1c (S-396) were significantly down-regulated by the treatment of xyloketal B (12.5, 25 and 50 μM) in HepG2 cells compared with that in the FFA group. In addition, xyloketal B treatment (12.5, 25, and 50 μM) of HepG2 cells reduced the protein expression of ACC1, ACL, FAS and up-regulated that of CPT-1A (Figure 5C), which is in accordance with the in vivo experimental results. These data indicated that xyloketal B ameliorates NAFLD through the de novo lipogenesis and fatty acid β-oxidation processes.

## 3. Discussion

NAFLD is the most common liver disease worldwide, and the secular increasing trend of NAFLD prevalence is alarming [26]. To date, there is no definite treatment available for NAFLD, and the pathogenesis of NAFLD is still not completely clear. Therefore, our study examined whether xyloketal B could improve NAFLD in both in vitro and in vivo models, and we also explored the possible mechanisms of action of xyloketal B, particularly SREBP-1c-mediated pathways.

Poor eating habits and an irrational diet structure are important factors leading to NAFLD. Previous studies have employed both in vivo and in vitro NAFLD models [27,28,29,30,31], and many studies have shown that fructose can lead to the occurrence of NAFLD [13,32,33,34]. In our study, the animal model was established using a high-fat and high-glucose diet with 10% fructose solution instead of drinking water. With this method, this model simulated the slow development of NAFLD which is similar to NAFLD induced by lifestyle. In our mouse modelgroup (8 weeks of a high-fat diet plus 10% fructose solution), the body weight, visceral fat weight, liver index, BMI, and serum levels of Glu, TC, CHOL, LDL-C, APOE, AST, and ALT were significantly elevated compared with those in the control group fed a normal diet. These results suggest that HFD+HFL feeding leads to hyperglycemia, obesity, hyperlipidemia, and liver injury. The liver histology showed that the livers of the model group had diffuse fat vacuoles and steatosis. In addition, higher levels of hepatic TG and CHOL also indicated excessive fat deposition in the livers. These results were consistent with the previously reported pathological features of non-alcoholic fatty liver. Thus, we determined that HFD+HFL-fed mice in this study developed typical NAFLD.

To ameliorate the disorders of hepatic histology and the physiological indexes of mice induced by HFD+HFL, we subjected the NAFLD mice to xyloketal B treatment for 40 days. During this experiment, daily diet consumption was stable and behavioral and physiological signs were normal, indicating that xyloketal B had no toxicity or influence on appetite. Treating mice with xyloketal B improved certain symptoms of NAFLD, decreasing the body weight, visceral fat weight, liver index, and BMI and reversing serum levels of Glu, TC, CHOL, LDL-C, APOE, AST, and ALT. Interestingly, the body of mice both in the HFD-HFL group and in the control group were increasing in a time-dependent manner. However, the changes in the body of mice fed with xyloketal were very small. The experimental animals have a lot of individual differences, although the mean body weight of 5 mg/kg/d Xyloketal group was the lowest among them, there was no significance among group C, group L, group Mi, group H, and group P (*p* > 0.05). According to all of the above-mentioned data, the possibility was greater that xyloketal B reduced lipogenesis and lipid accumulation to lessen the body weight. However, we could not exclude the possibility that xyloketal B may have induced a lack of appetite. We need more samples and further study to reveal the truth about xyloketal B influencing appetence. The H&E staining assay, hepatic TG content assay, and CHOL content assay results further suggested that xyloketal B significantly reduced lipid accumulation in the liver. This pharmacodynamic experiment demonstrates for the first time that xyloketal B significantly ameliorates the phenotypes of the HFD+HFL-induced NAFLD mouse model, and the curative effect was better than that of the atorvastatin positive control.

In vitro experiments were designed to confirm the lipid-lowering effect of xyloketal B. Our results indicate that xyloketal B reduces high levels of intracellular TG and alleviates lipid droplets in a HepG2 FFA-induced steatosis cell model in a dose-dependent manner, which is similar to the results reported previously [35,36]. In addition, our lab has also demonstrated that xyloketal B has anti-atherosclerotic effects on APOE-deficient mice, suggesting that it may be a potential treatment agent for atherosclerosis.

According to previous reports [37,38,39,40], the pathogeneses of NAFLD and atherosclerosis are both closely related to an imbalance of lipid metabolism. To explore the possible mechanism underlying the effectiveness of xyloketal B treatment of NAFLD, we investigated the effects of xyloketal B on lipid metabolism, mainly focusing on liver fatty acid synthesis and clearance pathways in vivo and in vitro.

SREBP-1c is the most important transcription factor regulating de novo lipogenesis in the liver, and it specifically regulates the expression of many types of key enzymes in the fatty acid biosynthesis pathway, including ACC1, FAS, and ACL. SREBP-1c levels are increased in the fatty livers of obese (ob/ob) mice [41] and in patients with histologically diagnosed NAFLD [42]. Thus, we investigated the relationship between SREBP-1c activation and xyloketal B in mouse model of NAFLD by Realtime-qPCR and Western blot analyses. The gene assay results showed that the mRNA expression of SREBP-1c and its downstream genes related to de novo lipogenesis (FAS, ACC1, SCD-1, and CD36) in the model group was significantly increased; however, xyloketal B treatment caused a significant down-regulation of these genes, suggesting that xyloketal B affects fatty acid synthesis by influencing SREBP-1c transcription levels to regulate the key genes of de novo lipogenesis. PEPCK, a key enzyme in gluconeogenesis, which catalyzes oxaloacetate to form phosphoenolpyruvic acid, consumes large amounts of acetyl coenzyme A, leads to a decrease of raw materials in the fatty acid synthesis pathway, and eventually causes the reduction of fatty acid synthesis and TG synthesis. In this study, the results showed that xyloketal B increased PEPCK mRNA expression, which in turn up-regulated gluconeogenesis, thus further demonstrating that PEPCK is a key enzyme in gluconeogenesis. CPT-1, the key rate-limiting and regulatory step in the process of β-oxidation, has two subtypes, CPT1A and CPT1B. CPT1 activates malonyl-CoA in the mitochondrial matrix to initiate β-oxidation. The experimental results also demonstrated that the mRNA expression of CPT1A and CPT1B was significantly up-regulated after xyloketal B treatment of mice with NAFLD. This result indicate that xyloketal B may enhance the β-oxidation of fatty acids by increasing the mRNA expression of CPT1, thus decreasing lipid accumulation and TG synthesis in the liver. UCP-2 is involved in metabolism disorders and oxidative stress, and it has also been demonstrated that the function of UCPs is to export fatty acids out of the mitochondria when there is an abundance of fatty acids present inside the matrix. Our results indicate that xyloketal B reduces UCP-2 mRNA expression, leading us to deduce that the effect of xyloketal B on NAFLD may be due to the down-regulation of UCP2 mRNA expression, These results are in accordance with previous studies [43,44].

Additionally, the protein levels were consistent with changes in the genetic levels. After xyloketal B intervention, the protein expression of SREBP-1c, ACC1, ACL, and FAS was significantly reduced, and the protein expression of CPT1A was significantly up-regulated.

We confirmed the potential mechanism in FFA-induced HepG2 cells. Our results show that xyloketal B reduced the levels of SREBP-1c, phosphorylated SREBP-1c (S-396) and its downstream proteins related to de novo lipogenesis. Interestingly, we found through dose escalation of xyloketal B that suppressive effect of a high dose on the level of SREBP-1c was more limited than the effect of a low or middle dose. We speculate that a high dose of xyloketal B might trigger other signaling pathways that can partly counteract the inhibitory effect of a low or middle dose on SREBP-1c expression. However, the mechanisms underlying the inverse relationship of the suppression of SREBP-1c with the xyloketal B concentration require further clarification. The up-regulation of CPT1A levels by xyloketal B suggest that xyloketal B beneficially regulates lipid metabolism by promoting β-oxidation. These data further confirm that xyloketal B reduces lipid accumulation via suppressing the SREBP-1c signaling pathway.

In conclusion, the present study reveals that xyloketal B reduces hepatic lipid accumulation. The mechanisms underlying the action of xyloketal B in NAFLD mainly involve the attenuation of lipid accumulation as well as the enhancement of gluconeogenesis and β-oxidation of fatty acids, suggesting that xyloketal B may be a potential drug for the treatment of NAFLD. Although our data indicate that the effect of xyloketal B is mediated by SREBP-1c, SREBP-1c seems not to be the target of xyloketal B. Based on the structure of xyloketal B, a number of nuclear receptors could be envisaged as its potential target; our future work will determine the specific target of xyloketal B and clarify the exact molecular mechanisms through which xyloketal B suppresses SREBP-1c.

## 4. Materials and Methods

### 4.1. Animals and Experimental Protocol

Male C57BL/6J mice weighing 18–22 g were purchased from Guangdong Medical Laboratory Animal Center (GDMLAC). All mice were 50–60 days old and were housed alone in standard cages for one week. The animals were housed in a controlled temperature of 24 ± 2 °C at 55%–60% relative humidity with 12 h light/dark cycles. The mice had free access to food and water ad libitum and were weighed twice a week throughout the experiment. According to the random number table generated, the animals were randomly divided into the following two groups receiving the following diets: (1) normal chow diet (control group, *n* = 29); and (2) high fat diet (HFD) plus 10% high fructose liquid (HFL) (model group, *n* = 90) for 8 weeks to induce NAFLD. Five mice from each group on the 40th day and three mice from each group on the 56th day were sacrificed to confirm the development of NAFLD. After the formation of NAFLD in mice, the model group animals were further divided into five groups based on body weight, as follows: low dose group (5 mg/kg/day xyloketal B via intraperitoneal injection), middle dose group (10 mg/kg/day xyloketal B via intraperitoneal injection), high dose group (20 mg/kg/day xyloketal B via intraperitoneal injection), and positive group (15 mg/kg/day atorvastatin via intraperitoneal injection). The animals were administered treatment once daily for 40 days. Blood was collected from the retro-orbital plexus using microcapillary tubes after overnight fasting on the 41st day. The blood was then centrifuged at 3000 rpm for 15 min. The animals were sacrificed by cervical dislocation, and tissues were rapidly removed. Among these tissues, peripheral adipose of the testis and liver were weighed. In addition, a portion of the liver was fixed in 4% paraformaldehyde for histopathological examination, and the remaining tissue was immediately frozen in liquid nitrogen or stored at −80 °C until further analysis. All experiments were conducted according to the guidelines for animal care and use of the People’s Republic of China and were approved by the animal ethics committee of the Chinese Academy of Medical Science (Beijing, People’s Republic of China).

### 4.2. Chemicals

Xyloketal B was synthesized at our organization. Sodium palmitate (PA), oleic acid (OA), fatostatin A, 3-(4,5-dimethylthiazol dimethylthiazol-2-yl)-2,5-diphenyltetrazolium bromide (MTT), and DMSO were purchased from Sigma (St. Louis, MO, USA). Dulbecco’s modified Eagle’s medium (DMEM), fetal bovine serum (FBS), penicillin, and streptomycin were purchased from Gibco Life Technologies (Carlsbad, CA, USA).

### 4.3. Assessment of Weight and Serum Biochemical Parameters

The liver, adipose, and body weight of mice were weighed after the mice were sacrificed, and the liver and body mass indexes were calculated. The fasting blood glucose, triglyceride (TG), total cholesterol (CHOL), alanine aminotransaminase (ALT), aspartate aminotransferase (AST), high-density lipoprotein cholesterol (HLDL-C), low-density lipoprotein cholesterol (LDL-C), and apolipoprotein E (APOE) levels in the serum were measured using a automatic biochemical analyzer (HITACHI7020, Tokyo, Japan).

### 4.4. Assessment of Liver TG and CHOL

Liver tissue (50–100 mg) was homogenized in nine volumes of ice-cold phosphate-buffered saline (PBS) solution (*v*/*w*) using an electric homogenate machine, followed by lipid extraction. TGs and CHOL were measured with kits (Nan Jing Jian Cheng Bioengineering Institute, Nanjing, China). Values were normalized to the protein concentration of liver homogenates as determined by a BCA assay (Beyotime Institute of Biotechnology, Shanghai, China).

### 4.5. Histological Analysis of Liver Tissue

Liver tissue samples were fixed in 4% paraformaldehyde, embedded in paraffin, sectioned (6-μm thickness), and stained with hematoxylin and eosin (H&E). Liver histology was performed using an inverted fluorescence microscope (OLYMPUS IX71, Tokyo, Japan).

### 4.6. Cell Culture and Treatment Protocols

HepG2 cells (Cell Bank, Chinese Academy of Science) were cultured in DMEM supplemented with 10% FBS, 100 U/mL penicillin, and 100 μg/mL streptomycin at 37 °C under a humidified atmosphere containing 5% CO_2_. Cells were used in experiments when they reached 80% confluence. Before treatment with xyloketal B, HepG2 cells were exposed to a free fatty acid (FFA) mixture (0.165 mMPA and 0.33 mMOA) for 24 h.

### 4.7. Cell Viability Assay

Cell survival was determined using an MTT assay kit. HepG2 cells (8 × 10^3^/well) were seeded in 96-well plates and incubated for 24 h, and the cells were then treated with different xyloketal B concentrations. MTT solution (20 μL) was added to each well, and the cells were incubated at 37 °C for 4 h. DMSO (150 μL) was then added, and the plates were measured at 490 nm.

### 4.8. Evaluation of Lipid Accumulation and TG Content

Lipid accumulation was evaluated by Oil Red O staining. HepG2 cells were washed with sterile PBS and fixed with 10% formalin in PBS (pH = 7.4) for 30 min. The cells were then washed three times with sterile PBS and incubated with diluted Oil Red O reagent for 10 min. The cells were washed three times with sterile PBS and imaged using light microscopy (B-POL, Chongqing, China). After imaging, the dye was eluted with 100% isopropanol and quantified by measuring the optical absorbance at 540 nm. The TG concentration in HepG2 cells was measured using a commercial kit (Nan Jing Jian Cheng Bioengineering Institute, Nanjing, China) according to the manufacturer’s protocol. The protein concentrations were measured using a BCA assay.

### 4.9. RNA Isolation, Reverse Transcription, and Real-Time PCR

Total RNA from frozen liver tissue samples was isolated in accordance with the protocol of the RNeasy Plus Mini Kit (Qiagen, Hilden, Germany). Extracted RNA was converted into cDNA by reverse transcription with the iScript™ cDNA Synthesis Kit (Bio-Rad, Hercules, CA, USA). cDNA was used as a template to quantify specific gene expression by real-time PCR using SYBR Green (CFX96 Touch™ fluorescence quantitative PCR detection system, Bio-Rad). The mRNA levels were analyzed for SREBP1-c, FAS, ACC1, CPT1-1A, CPT1-1B, SCD, UCP-2, and PEPCK in the liver tissue, and the relative expression of the target genes was compared after normalization against glyceraldehyde-3-phosphate dehydrogenase (GAPDH). The primers for specific genes and GAPDH are listed in Table 2.

### 4.10. Western Blot Analysis

Liver tissues were homogenized in RIPA lysis buffer (P0013B, Beyotime) containing a phosphatase and protease inhibitor mix (Roche). Total protein was obtained by centrifugation at 13,000 rpm for 40 min at 4 °C, and the total protein was quantified using the BCA protein Assay Kit (Beyotime). The supernatants (30 μg protein per well) were separated by 8% or 12% sodium dodecyl sulfate-polyacrylamide gel electrophoresis (SDS-PAGE) and transferred to a polyvinylidene fluoride (PVDF) membrane. The membranes were incubated with the following antibodies: FAS (Cell Signaling Technology), ACL (Cell Signaling Technology), ACC1 (Cell Signaling Technology), CPT-1A (Protein Tech), SREBP-1C (Abcam), p-SREBP-1C-(S-396) (Abcam), and GAPDH (Cell Signaling Technology). The membranes were washed with TBST buffer and incubated with secondary antibodies. All the bands were visualized using chemiluminescence. To ensure equal loading, all the proteins were normalized to GAPDH.

### 4.11. Statistical Analysis

All results are expressed as the mean ±SEM. The calculations were performed and the graphs were generated using Graph-Pad Prism 5 software (Graph-Pad Software, La Jolla, CA, USA). Statistical comparisons were performed using unpaired one-way analysis of variance (ANOVA) or the two-tailed unpaired Student’s *t*-test. The results were considered significantly different at <0.05.

## Figures and Tables

**Table 1:** Weight-related parameters and serum biochemical levels in mice.

Parameter	Control	HFD+HFL	5 mg/kg/d Xyloketal B	10 mg/kg/d Xyloketal B	20 mg/kg/d Xyloketal B	15 mg/kg/d Atorvastatin
Body weight (g)	21.84 ± 0.74 ^b^	31.92 ± 0.37	24.25 ± 0.65 ^b^	26.44 ± 0.94 ^b^	26.34 ± 0.44 ^b^	26.04 ± 0.80 ^b^
Liver/body weight (%)	4.40 ± 0.10 ^b^	5.38 ± 0.12	3.83 ± 0.23 ^b^	3.65 ± 0.24 ^b^	3.81 ± 0.09 ^b^	3.80 ± 0.11 ^b^
Body weight/height (%)	0.77 ± 0.02 ^b^	1.12 ± 0.02	0.85 ± 0.02 ^b^	0.95 ± 0.05 ^b^	0.94 ± 0.02 ^b^	0.90 ± 0.03 ^b^
Visceral fat (g)	0.49 ± 0.74 ^b^	1.11 ± 0.37	0.73 ± 0.65 ^b^	0.69 ± 0.94 ^b^	0.51 ± 0.44 ^b^	0.35 ± 0.80 ^b^
Glucose (mmol/L)	6.87 ± 0.53 ^a^	11.00 ± 0.57	7.33 ± 0.91 ^a^	7.64 ± 0.41 ^a^	7.83 ± 0.73 ^a^	9.98 ± 0.64
ALT (mmol/L)	35.50 ± 2.11 ^a^	43.80 ± 2.40	29.25 ± 1.21 ^b^	30.88 ± 1.23 ^b^	32.38 ± 1.50 ^b^	35.29 ± 2.00 ^a^
AST (mmol/L)	118.40 ± 3.12 ^a^	131.70 ± 5.79	110.17 ± 5.84 ^a^	115.00 ± 6.26	125.40 ± 7.26	110.25 ± 2.87 ^a^
Triglyceride (mmol/L)	0.96 ± 0.02 ^a^	1.26 ± 0.10	0.71 ± 0.02 ^b^	0.73 ± 0.07 ^b^	0.77 ± 0.04 ^b^	0.78 ± 0.01 ^b^
Cholesterol (mmol/L)	1.97 ± 0.06 ^b^	3.95 ± 0.32	2.22 ± 0.34 ^b^	2.55 ± 0.25 ^b^	2.53 ± 0.14 ^b^	1.87 ± 0.19 ^b^
LDL cholesterol (mmol/L)	0.10 ± 0.01 ^b^	0.87 ± 0.04	0.55 ± 0.07 ^b^	0.50 ± 0.09 ^b^	0.37 ± 0.04 ^b^	0.32 ± 0.03 ^b^
HDL cholesterol (mmol/L)	1.20 ± 0.03 ^a^	1.56 ± 0.10	1.36 ± 0.07 ^a^	1.54 ± 0.07	1.52 ± 0.08	1.65 ± 0.06
APOE (mmol/L)	12.53 ± 0.55 ^b^	3.17 ± 0.88	8.53 ± 1.28	17.49 ± 1.05 ^b^	16.60 ± 1.74 ^b^	4.71 ± 1.13

Data are expressed as the means ± SEM (*n* = 8), ^a^ *p* < 0.05, ^b^ *p* < 0.001 compared with mice fed the HFD+HFL.

**Table 2:** Primer sequences used for real-time Q-PCR.

Gene	Forward Primers	Reverse Primers
SREBP	CCCTGTGTACGGCCTTT	TTGCGATGTCTCCAGAGTG
ACC1	AAGTCCTTGGTCGGGAAGTATACA	ACTCCCTCAAAGTCATCACAAACA
FAS	TGGTGAATTGTCTCCGAAAAGA	CACGTTCATCACGAGGTCATG
SCD-1	TTCTTACACGACCACCACCA	CCGAAGAGGCAGGTGTAGAG
CD36	TTGAAGGCATTCCCACGTATC	CGGACCCGTTGGCAAA
PEPCK	AAGCATTCAACGCCAGGTTC	GGGCGAGTCTGTCAGTTCAAT
CPT1A	GCACTGCAGCTCCCACATTACAA	CTCAGACAGTACCTCCTTCAGGAAA
CPT1B	CTCCGCCTGAGCCATGAAG	CACCAGTATGATGCCATTCT
UCP-2	CCGCATTGGCCTCTACGACTC	GGAGCATGGTCGGGCACAGT
GAPDH	CCTTCCGTGTTCCTACCC	CCCAAGATGCCCTTCAGT
